# 3-[1-(4-Isobutyl­phen­yl)eth­yl]-6-(4-methyl­phen­yl)-1,2,4-triazolo[3,4-*b*][1,3,4]thia­diazole

**DOI:** 10.1107/S1600536808013883

**Published:** 2008-05-14

**Authors:** Hoong-Kun Fun, Samuel Robinson Jebas, Ibrahim Abdul Razak, K. V. Sujith, P. S. Patil, B. Kalluraya, S. M. Dharmaprakash

**Affiliations:** a X-ray Crystallography Unit, School of Physics, Universiti Sains Malaysia, 11800 USM, Penang, Malaysia; b Department of Studies in Chemistry, Mangalore University, Mangalagangotri, Mangalore 574 199, India; c Department of Studies in Physics, Mangalore University, Mangalagangotri, Mangalore 574 199, India

## Abstract

In the title compound, C_22_H_24_N_4_S, the methylphenyl and isobutylphenyl rings are inclined at an angle of 79.98 (1)° and they form dihedral angles of 4.59 (1) and 75.47 (1)°, respectively, with the triazolothia­diazole unit. An intra­molecular C—H⋯S hydrogen bond generates an *S*(5) ring motif. The crystal structure is stabilized by inter­molecular C—H⋯N hydrogen bonds and weak C—H⋯π and π–π inter­actions [centroid–centroid distances between the thia­diazole ring and a symmetry-related phenyl ring and between the triazole ring and the phenyl ring range from 3.5680 (8) to 3.7313 (8) Å].

## Related literature

For information on the biological activity of triazole derivatives, thia­diazo­les and triazolothia­diazole compounds, see: Holla *et al.* (2003 ▶); Bekircan & Bektas (2006 ▶); Zhou *et al.* (2007 ▶); Bhat *et al.* (2004 ▶); Mathew *et al.* (2007 ▶); Karthikeyan *et al.* (2007 ▶); Chaturvedi *et al.* (1988 ▶); Shawali & Sayed (2006 ▶). For details of hydrogen-bond motifs, see: Bernstein *et al.* (1995 ▶). For bond-length data, see: Allen *et al.* (1987 ▶). For related literature, see: Tayseer *et al.* (2002 ▶).
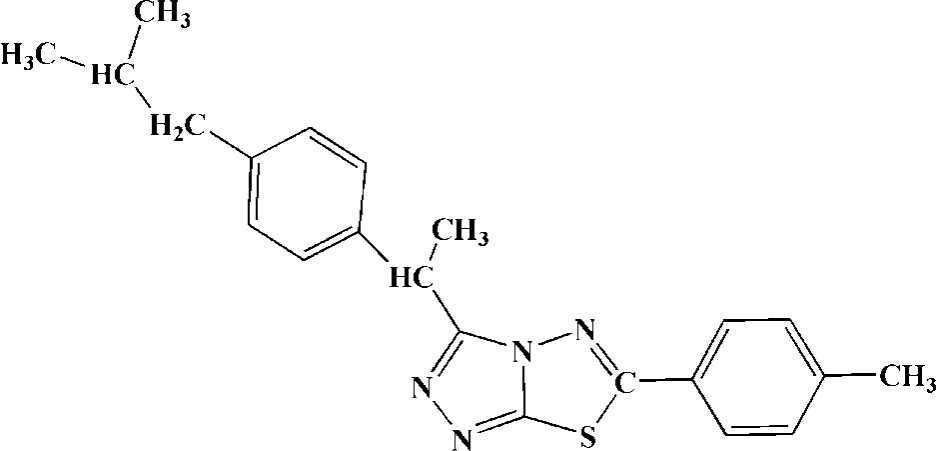


## Experimental

### 

#### Crystal data

C_22_H_24_N_4_S
*M* *_r_* = 376.51
Triclinic, 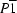

*a* = 7.2545 (1) Å
*b* = 8.1764 (1) Å
*c* = 17.6556 (3) Å
α = 97.390 (1)°
β = 96.120 (1)°
γ = 106.240 (1)°
*V* = 984.90 (2) Å^3^
*Z* = 2
Mo *K*α radiation
μ = 0.18 mm^−1^
*T* = 100.0 (1) K
0.46 × 0.20 × 0.18 mm

#### Data collection

Bruker SMART APEXII CCD area-detector diffractometer
Absorption correction: multi-scan (*SADABS*; Bruker, 2005 ▶) *T*_min_ = 0.922, *T*_max_ = 0.969
15868 measured reflections
5687 independent reflections
4391 reflections with *I* > 2σ(*I*)
*R*_int_ = 0.029

#### Refinement

*R*[*F* ^2^ > 2σ(*F* ^2^)] = 0.047
*wR*(*F* ^2^) = 0.141
*S* = 1.08
5687 reflections
248 parameters
H-atom parameters constrained
Δρ_max_ = 0.45 e Å^−3^
Δρ_min_ = −0.39 e Å^−3^

### 

Data collection: *APEX2* (Bruker, 2005 ▶); cell refinement: *APEX2*; data reduction: *SAINT* (Bruker, 2005 ▶); program(s) used to solve structure: *SHELXTL* (Sheldrick, 2008 ▶); program(s) used to refine structure: *SHELXTL*; molecular graphics: *SHELXTL*; software used to prepare material for publication: *SHELXTL* and *PLATON* (Spek, 2003 ▶).

## Supplementary Material

Crystal structure: contains datablocks global, I. DOI: 10.1107/S1600536808013883/sj2495sup1.cif

Structure factors: contains datablocks I. DOI: 10.1107/S1600536808013883/sj2495Isup2.hkl

Additional supplementary materials:  crystallographic information; 3D view; checkCIF report

## Figures and Tables

**Table 1:** Hydrogen-bond geometry (Å, °)

*D*—H⋯*A*	*D*—H	H⋯*A*	*D*⋯*A*	*D*—H⋯*A*
C5—H5*A*⋯S1	0.93	2.70	3.1194 (16)	108
C15—H15*A*⋯N3^i^	0.93	2.48	3.343 (2)	155
C4—H4*A*⋯*Cg*1^ii^	0.93	2.62	3.5063	160

Symmetry codes: (i) 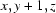; (ii) 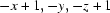. *Cg*1 is the centroid of the C11–C16 ring.

**Fig. 1.**
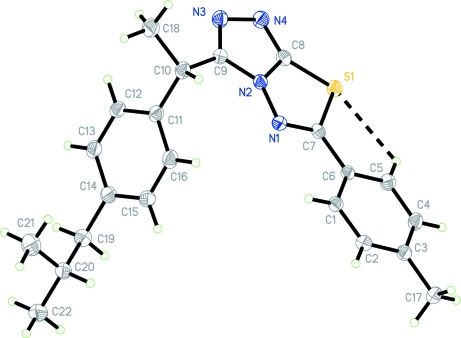
The molecular structure of the title compound, showing 50% probability displacement ellipsoids and the atom-numbering scheme. Dashed lines indicate hydrogen bonds.

**Fig. 2.**
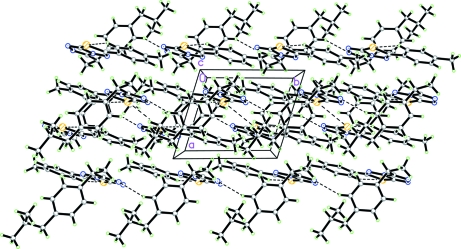
The crystal packing of the title compound, viewed along the c axis. Hydrogen bonds are shown as dashed lines.

## supplementary crystallographic information

### Comment

Triazoles and their heterocyclic derivatives represent an interesting class of compounds possessing a wide spectrum of biological activity, such as anticancer, anticonvulsant, analgesic, antibacterial, anthelmintic, antitubercular and anti-inflammatory activities (Holla *et al.*, 2003; Bekircan & Bektas, 2006; Zhou *et al.*,  2007). Similarly 1, 3,4-thiadiazoles were also found to possess antitumor, anti-inflammatory, antibacterial, antifungal, anticonvulsant and antitubercular properties (Bhat *et al.*, 2004; Mathew *et al.*,  2007). Thus triazolothiadiazole systems may be viewed as cyclic analogues of two very important components, which often display diverse pharmacological properties. Triazolothiadiazoles obtained by fusing the biolabile 1,2,4-triazole and 1,3,4-thiadiazole rings together have been reported to possess similar biological properties (Karthikeyan *et al.*, 2007; Chaturvedi *et al.*,  1988; Shawali & Sayed 2006) and the crystal structure of the title triazolothiadiazole compound is reported here.

Bond lengths and angles in the title compound (Fig 1) have normal values (Allen *et al.*, 1987). The triazolothiadiazole ring is planar with the maximum deviation of 0.016 (2)Å for atom C7. The planes through the C1—C6 and C11—C16 rings form dihedral angles of 4.59 (1)° and 75.47 (1)° respectively, with the triazolothiadiazole unit. This is also planar with a dihedral angle of 1.34 (2) °  between the two five membered rings. A weak intramolecular C—H···S hydrogen bond generates an S(5) ring motif (Bernstein *et al.*, (1995) and contributes to the planarity of the 4-methylphenyl-triazolothiadiazole portion of the molecule.

The crystal packing is stabilized by intermolecular C—H···N hydrogen bonds and a weak C—H···π interaction involving the C11—C16 ring (centroid Cg1, Table 1). π–π interactions are observed between the thiadiazole ring (S1/C7/N1—N2/C8) and the symmetry related phenyl rings (C1—C6) and between the triazole ring and the phenyl ring (C1—C6) with centroid to centroid distances ranging from 3.5680 (8)–3.7313 (8)Å [symmetry codes:1-*X*,-Y,1-*Z*;2-*X*, -Y,1-*Z*].

### Experimental

A mixture of 4-amino-3-mercapto-5-[1-(4-isobutylphenyl)ethyl]-1, 2,4-triazole (0.01 mol), *p*-toluic acid (0.01 mol) and 10 ml POCl_3_ was refluxed on a water bath for about 9 h. Excess of POCl_3_ was removed under reduced pressure. The reaction mixture was cooled, poured into crushed ice, and neutralized with aqueous ammonia. The resulting solid product was filtered off, washed with water, dried, and recrystallized from a mixture of ethanol and dimethylformamide, 1/1, v/v. (Yield 61%; m.p. 134–1360 C). Analysis (%) for C_22_H_24_N_4_S found (calculated): C 70.16 (70.21), H 6.31 (6.38), N 14.78 (14.89).

### Refinement

H atoms were positioned geometrically [C–H = 0.93–0.98 Å] and refined using a riding model, with *U*_iso_(H) = -1.2 to -1.5*U*_eq_(C). A rotating-group model was used for the methyl groups.

### Crystal data

**Table:**

C_22_H_24_N_4_S	*Z* = 2
*M**_r_* = 376.51	*F*_000_ = 400
Triclinic, *P*1	*D*_x_ = 1.270 Mg m^−^^3^
Hall symbol: -P 1	Mo *K*α radiation λ = 0.71073 Å
*a* = 7.2545 (1) Å	Cell parameters from 5597 reflections
*b* = 8.1764 (1) Å	θ = 2.6–32.0º
*c* = 17.6556 (3) Å	µ = 0.18 mm^−^^1^
α = 97.539 (1)º	*T* = 100.0 (1) K
β = 96.712 (1)º	Block, colourless
γ = 106.024 (1)º	0.46 × 0.20 × 0.18 mm
*V* = 984.90 (2) Å^3^	

### Data collection

**Table:**

Bruker SMART APEXII CCD area-detector diffractometer	5687 independent reflections
Radiation source: fine-focus sealed tube	4391 reflections with *I* > 2σ(*I*)
Monochromator: graphite	*R*_int_ = 0.029
*T* = 100.0(1) K	θ_max_ = 30.0º
φ and ω scans	θ_min_ = 1.2º
Absorption correction: multi-scan(SADABS; Bruker, 2005)	*h* = −10→8
*T*_min_ = 0.922, *T*_max_ = 0.969	*k* = −11→11
15868 measured reflections	*l* = −24→24

### Refinement

**Table:**

Refinement on *F*^2^	Secondary atom site location: difference Fourier map
Least-squares matrix: full	Hydrogen site location: inferred from neighbouring sites
*R*[*F*^2^ > 2σ(*F*^2^)] = 0.047	H-atom parameters constrained
*wR*(*F*^2^) = 0.142	*w* = 1/[σ^2^(*F*_o_^2^) + (0.0792*P*)^2^ + 0.0947*P*] where *P* = (*F*_o_^2^ + 2*F*_c_^2^)/3
*S* = 1.08	(Δ/σ)_max_ = 0.001
5687 reflections	Δρ_max_ = 0.45 e Å^−^^3^
248 parameters	Δρ_min_ = −0.38 e Å^−^^3^
Primary atom site location: structure-invariant direct methods	Extinction correction: none

### Special details

**Table:**

Experimental. The data was collected with the Oxford Cyrosystem Cobra low-temperature attachment.
Geometry. All e.s.d.'s (except the e.s.d. in the dihedral angle between two l.s. planes) are estimated using the full covariance matrix. The cell e.s.d.'s are taken into account individually in the estimation of e.s.d.'s in distances, angles and torsion angles; correlations between e.s.d.'s in cell parameters are only used when they are defined by crystal symmetry. An approximate (isotropic) treatment of cell e.s.d.'s is used for estimating e.s.d.'s involving l.s. planes.
Refinement. Refinement of *F*^2^ against ALL reflections. The weighted *R*-factor *wR* and goodness of fit *S* are based on *F*^2^, conventional *R*-factors *R* are based on *F*, with *F* set to zero for negative *F*^2^. The threshold expression of *F*^2^ > σ(*F*^2^) is used only for calculating *R*-factors(gt) *etc*. and is not relevant to the choice of reflections for refinement. *R*-factors based on *F*^2^ are statistically about twice as large as those based on *F*, and *R*- factors based on ALL data will be even larger.

### Fractional atomic coordinates and isotropic or equivalent isotropic displacement parameters (Å^2^)

**Table:**

	*x*	*y*	*z*	*U*_iso_*/*U*_eq_	
S1	0.65630 (5)	0.70616 (5)	0.50456 (2)	0.02030 (11)	
N1	0.77613 (18)	0.91957 (15)	0.40935 (7)	0.0187 (3)	
N2	0.73966 (17)	0.75288 (15)	0.37235 (7)	0.0177 (3)	
N3	0.6927 (2)	0.50811 (16)	0.29652 (7)	0.0237 (3)	
N4	0.6461 (2)	0.47006 (16)	0.36878 (7)	0.0236 (3)	
C1	0.8328 (2)	1.23186 (19)	0.51493 (8)	0.0197 (3)	
H1A	0.8673	1.2404	0.4662	0.024*	
C2	0.8563 (2)	1.3798 (2)	0.56768 (9)	0.0218 (3)	
H2A	0.9089	1.4871	0.5541	0.026*	
C3	0.8029 (2)	1.3718 (2)	0.64094 (8)	0.0212 (3)	
C4	0.7242 (2)	1.2089 (2)	0.65980 (9)	0.0237 (3)	
H4A	0.6859	1.2004	0.7080	0.028*	
C5	0.7022 (2)	1.0597 (2)	0.60774 (8)	0.0222 (3)	
H5A	0.6505	0.9524	0.6214	0.027*	
C6	0.7572 (2)	1.06958 (18)	0.53493 (8)	0.0174 (3)	
C7	0.7360 (2)	0.91381 (18)	0.47910 (8)	0.0172 (3)	
C8	0.6765 (2)	0.62112 (18)	0.41226 (8)	0.0193 (3)	
C9	0.7476 (2)	0.67599 (19)	0.29953 (8)	0.0202 (3)	
C10	0.8042 (2)	0.7724 (2)	0.23493 (9)	0.0233 (3)	
H10A	0.9227	0.8674	0.2557	0.028*	
C11	0.6460 (2)	0.85129 (19)	0.20767 (8)	0.0220 (3)	
C12	0.4859 (2)	0.75782 (19)	0.15229 (8)	0.0228 (3)	
H12A	0.4751	0.6447	0.1309	0.027*	
C13	0.3422 (2)	0.8308 (2)	0.12848 (9)	0.0245 (3)	
H13A	0.2379	0.7664	0.0907	0.029*	
C14	0.3510 (2)	0.99849 (19)	0.15996 (9)	0.0251 (3)	
C15	0.5107 (3)	1.0916 (2)	0.21594 (9)	0.0289 (4)	
H15A	0.5197	1.2035	0.2384	0.035*	
C16	0.6563 (3)	1.0200 (2)	0.23864 (9)	0.0274 (4)	
H16A	0.7628	1.0857	0.2752	0.033*	
C17	0.8301 (3)	1.5347 (2)	0.69710 (9)	0.0274 (3)	
H17A	0.7632	1.5074	0.7397	0.041*	
H17B	0.7783	1.6128	0.6715	0.041*	
H17C	0.9661	1.5879	0.7160	0.041*	
C18	0.8520 (3)	0.6536 (2)	0.17039 (9)	0.0289 (4)	
H18A	0.9693	0.6281	0.1885	0.043*	
H18B	0.8694	0.7101	0.1263	0.043*	
H18C	0.7472	0.5481	0.1560	0.043*	
C19	0.1932 (3)	1.0768 (2)	0.13430 (10)	0.0296 (4)	
H19A	0.1290	1.0994	0.1780	0.036*	
H19B	0.0973	0.9928	0.0947	0.036*	
C20	0.2641 (3)	1.2447 (2)	0.10233 (10)	0.0312 (4)	
H20A	0.3529	1.3316	0.1440	0.037*	
C21	0.3738 (3)	1.2215 (3)	0.03589 (11)	0.0437 (5)	
H21A	0.4838	1.1846	0.0532	0.066*	
H21B	0.4176	1.3293	0.0178	0.066*	
H21C	0.2897	1.1360	−0.0055	0.066*	
C22	0.0920 (3)	1.3099 (2)	0.07796 (11)	0.0419 (5)	
H22A	0.1372	1.4156	0.0584	0.063*	
H22B	0.0298	1.3303	0.1219	0.063*	
H22C	0.0008	1.2249	0.0383	0.063*	

### Atomic displacement parameters (Å^2^)

**Table:**

	*U*^11^	*U*^22^	*U*^33^	*U*^12^	*U*^13^	*U*^23^
S1	0.0225 (2)	0.02041 (19)	0.01790 (18)	0.00448 (15)	0.00473 (14)	0.00591 (13)
N1	0.0200 (6)	0.0182 (6)	0.0187 (6)	0.0067 (5)	0.0031 (5)	0.0038 (5)
N2	0.0192 (6)	0.0172 (6)	0.0168 (6)	0.0051 (5)	0.0027 (5)	0.0044 (4)
N3	0.0277 (7)	0.0225 (6)	0.0202 (6)	0.0061 (6)	0.0036 (5)	0.0041 (5)
N4	0.0282 (7)	0.0208 (6)	0.0214 (6)	0.0052 (5)	0.0053 (5)	0.0059 (5)
C1	0.0205 (7)	0.0228 (7)	0.0177 (7)	0.0083 (6)	0.0047 (6)	0.0045 (6)
C2	0.0218 (7)	0.0214 (7)	0.0231 (7)	0.0077 (6)	0.0035 (6)	0.0047 (6)
C3	0.0189 (7)	0.0263 (7)	0.0194 (7)	0.0101 (6)	0.0011 (6)	0.0018 (6)
C4	0.0258 (8)	0.0294 (8)	0.0172 (7)	0.0096 (7)	0.0051 (6)	0.0039 (6)
C5	0.0250 (8)	0.0234 (7)	0.0194 (7)	0.0071 (6)	0.0051 (6)	0.0061 (6)
C6	0.0153 (7)	0.0208 (7)	0.0169 (6)	0.0073 (6)	0.0012 (5)	0.0031 (5)
C7	0.0146 (6)	0.0197 (7)	0.0180 (7)	0.0052 (5)	0.0019 (5)	0.0056 (5)
C8	0.0185 (7)	0.0195 (7)	0.0201 (7)	0.0043 (6)	0.0035 (6)	0.0064 (5)
C9	0.0219 (7)	0.0204 (7)	0.0177 (7)	0.0067 (6)	0.0016 (6)	0.0020 (5)
C10	0.0273 (8)	0.0236 (7)	0.0187 (7)	0.0064 (6)	0.0051 (6)	0.0040 (6)
C11	0.0302 (8)	0.0198 (7)	0.0163 (6)	0.0055 (6)	0.0069 (6)	0.0057 (5)
C12	0.0314 (8)	0.0183 (7)	0.0189 (7)	0.0062 (6)	0.0071 (6)	0.0032 (5)
C13	0.0310 (8)	0.0214 (7)	0.0189 (7)	0.0045 (6)	0.0039 (6)	0.0031 (6)
C14	0.0357 (9)	0.0217 (7)	0.0201 (7)	0.0095 (7)	0.0065 (7)	0.0072 (6)
C15	0.0447 (10)	0.0174 (7)	0.0231 (8)	0.0091 (7)	0.0002 (7)	0.0030 (6)
C16	0.0370 (9)	0.0202 (7)	0.0205 (7)	0.0032 (7)	−0.0003 (7)	0.0028 (6)
C17	0.0321 (9)	0.0292 (8)	0.0207 (7)	0.0132 (7)	0.0005 (7)	−0.0020 (6)
C18	0.0312 (9)	0.0353 (9)	0.0238 (8)	0.0140 (7)	0.0078 (7)	0.0060 (7)
C19	0.0377 (9)	0.0277 (8)	0.0259 (8)	0.0134 (7)	0.0049 (7)	0.0053 (6)
C20	0.0482 (11)	0.0217 (7)	0.0235 (8)	0.0140 (8)	−0.0024 (7)	0.0024 (6)
C21	0.0660 (14)	0.0411 (11)	0.0307 (9)	0.0211 (10)	0.0107 (9)	0.0158 (8)
C22	0.0616 (13)	0.0302 (9)	0.0340 (10)	0.0234 (9)	−0.0081 (9)	−0.0010 (7)

### Geometric parameters (Å, °)

**Table:**

S1—C8	1.7248 (15)	C12—C13	1.388 (2)
S1—C7	1.7714 (14)	C12—H12A	0.9300
N1—C7	1.3018 (18)	C13—C14	1.391 (2)
N1—N2	1.3716 (17)	C13—H13A	0.9300
N2—C8	1.3672 (18)	C14—C15	1.395 (2)
N2—C9	1.3699 (18)	C14—C19	1.511 (2)
N3—C9	1.3113 (19)	C15—C16	1.387 (2)
N3—N4	1.4092 (18)	C15—H15A	0.9300
N4—C8	1.3133 (19)	C16—H16A	0.9300
C1—C2	1.383 (2)	C17—H17A	0.9600
C1—C6	1.397 (2)	C17—H17B	0.9600
C1—H1A	0.9300	C17—H17C	0.9600
C2—C3	1.397 (2)	C18—H18A	0.9600
C2—H2A	0.9300	C18—H18B	0.9600
C3—C4	1.397 (2)	C18—H18C	0.9600
C3—C17	1.503 (2)	C19—C20	1.533 (2)
C4—C5	1.386 (2)	C19—H19A	0.9700
C4—H4A	0.9300	C19—H19B	0.9700
C5—C6	1.395 (2)	C20—C21	1.513 (3)
C5—H5A	0.9300	C20—C22	1.527 (3)
C6—C7	1.464 (2)	C20—H20A	0.9800
C9—C10	1.503 (2)	C21—H21A	0.9600
C10—C11	1.525 (2)	C21—H21B	0.9600
C10—C18	1.533 (2)	C21—H21C	0.9600
C10—H10A	0.9800	C22—H22A	0.9600
C11—C12	1.392 (2)	C22—H22B	0.9600
C11—C16	1.394 (2)	C22—H22C	0.9600
			
C8—S1—C7	87.77 (7)	C12—C13—H13A	119.3
C7—N1—N2	107.74 (12)	C14—C13—H13A	119.3
C8—N2—C9	105.94 (12)	C13—C14—C15	117.59 (15)
C8—N2—N1	118.63 (12)	C13—C14—C19	121.14 (15)
C9—N2—N1	135.41 (12)	C15—C14—C19	121.27 (14)
C9—N3—N4	109.48 (12)	C16—C15—C14	121.08 (14)
C8—N4—N3	104.93 (12)	C16—C15—H15A	119.5
C2—C1—C6	119.97 (13)	C14—C15—H15A	119.5
C2—C1—H1A	120.0	C15—C16—C11	121.19 (15)
C6—C1—H1A	120.0	C15—C16—H16A	119.4
C1—C2—C3	121.54 (14)	C11—C16—H16A	119.4
C1—C2—H2A	119.2	C3—C17—H17A	109.5
C3—C2—H2A	119.2	C3—C17—H17B	109.5
C4—C3—C2	118.01 (14)	H17A—C17—H17B	109.5
C4—C3—C17	121.60 (14)	C3—C17—H17C	109.5
C2—C3—C17	120.39 (14)	H17A—C17—H17C	109.5
C5—C4—C3	120.94 (14)	H17B—C17—H17C	109.5
C5—C4—H4A	119.5	C10—C18—H18A	109.5
C3—C4—H4A	119.5	C10—C18—H18B	109.5
C4—C5—C6	120.46 (14)	H18A—C18—H18B	109.5
C4—C5—H5A	119.8	C10—C18—H18C	109.5
C6—C5—H5A	119.8	H18A—C18—H18C	109.5
C5—C6—C1	119.07 (13)	H18B—C18—H18C	109.5
C5—C6—C7	121.42 (13)	C14—C19—C20	114.77 (15)
C1—C6—C7	119.50 (12)	C14—C19—H19A	108.6
N1—C7—C6	122.53 (13)	C20—C19—H19A	108.6
N1—C7—S1	116.63 (11)	C14—C19—H19B	108.6
C6—C7—S1	120.84 (10)	C20—C19—H19B	108.6
N4—C8—N2	111.28 (13)	H19A—C19—H19B	107.6
N4—C8—S1	139.48 (11)	C21—C20—C22	111.13 (15)
N2—C8—S1	109.21 (10)	C21—C20—C19	111.68 (14)
N3—C9—N2	108.36 (13)	C22—C20—C19	109.77 (16)
N3—C9—C10	127.27 (13)	C21—C20—H20A	108.0
N2—C9—C10	124.35 (13)	C22—C20—H20A	108.0
C9—C10—C11	109.88 (13)	C19—C20—H20A	108.0
C9—C10—C18	109.90 (13)	C20—C21—H21A	109.5
C11—C10—C18	113.81 (13)	C20—C21—H21B	109.5
C9—C10—H10A	107.7	H21A—C21—H21B	109.5
C11—C10—H10A	107.7	C20—C21—H21C	109.5
C18—C10—H10A	107.7	H21A—C21—H21C	109.5
C12—C11—C16	117.76 (15)	H21B—C21—H21C	109.5
C12—C11—C10	121.62 (13)	C20—C22—H22A	109.5
C16—C11—C10	120.61 (14)	C20—C22—H22B	109.5
C13—C12—C11	120.99 (14)	H22A—C22—H22B	109.5
C13—C12—H12A	119.5	C20—C22—H22C	109.5
C11—C12—H12A	119.5	H22A—C22—H22C	109.5
C12—C13—C14	121.37 (15)	H22B—C22—H22C	109.5
			
C7—N1—N2—C8	−1.13 (17)	N4—N3—C9—N2	0.11 (17)
C7—N1—N2—C9	177.66 (15)	N4—N3—C9—C10	−178.01 (14)
C9—N3—N4—C8	−0.02 (17)	C8—N2—C9—N3	−0.16 (16)
C6—C1—C2—C3	−1.1 (2)	N1—N2—C9—N3	−179.05 (14)
C1—C2—C3—C4	−0.1 (2)	C8—N2—C9—C10	178.03 (14)
C1—C2—C3—C17	−179.98 (13)	N1—N2—C9—C10	−0.9 (3)
C2—C3—C4—C5	0.9 (2)	N3—C9—C10—C11	108.16 (17)
C17—C3—C4—C5	−179.20 (14)	N2—C9—C10—C11	−69.68 (18)
C3—C4—C5—C6	−0.5 (2)	N3—C9—C10—C18	−17.8 (2)
C4—C5—C6—C1	−0.6 (2)	N2—C9—C10—C18	164.34 (14)
C4—C5—C6—C7	179.78 (13)	C9—C10—C11—C12	−85.94 (17)
C2—C1—C6—C5	1.4 (2)	C18—C10—C11—C12	37.8 (2)
C2—C1—C6—C7	−178.96 (13)	C9—C10—C11—C16	93.15 (17)
N2—N1—C7—C6	−179.59 (12)	C18—C10—C11—C16	−143.13 (15)
N2—N1—C7—S1	1.19 (15)	C16—C11—C12—C13	0.4 (2)
C5—C6—C7—N1	176.10 (14)	C10—C11—C12—C13	179.48 (13)
C1—C6—C7—N1	−3.5 (2)	C11—C12—C13—C14	−1.2 (2)
C5—C6—C7—S1	−4.72 (19)	C12—C13—C14—C15	0.6 (2)
C1—C6—C7—S1	175.70 (11)	C12—C13—C14—C19	−179.48 (14)
C8—S1—C7—N1	−0.81 (12)	C13—C14—C15—C16	0.7 (2)
C8—S1—C7—C6	179.96 (12)	C19—C14—C15—C16	−179.20 (15)
N3—N4—C8—N2	−0.09 (16)	C14—C15—C16—C11	−1.5 (3)
N3—N4—C8—S1	178.05 (14)	C12—C11—C16—C15	0.9 (2)
C9—N2—C8—N4	0.15 (17)	C10—C11—C16—C15	−178.18 (14)
N1—N2—C8—N4	179.27 (12)	C13—C14—C19—C20	−122.58 (16)
C9—N2—C8—S1	−178.56 (10)	C15—C14—C19—C20	57.3 (2)
N1—N2—C8—S1	0.55 (16)	C14—C19—C20—C21	55.9 (2)
C7—S1—C8—N4	−178.04 (19)	C14—C19—C20—C22	179.58 (14)
C7—S1—C8—N2	0.12 (11)		

### Hydrogen-bond geometry (Å, °)

**Table:**

*D*—H···*A*	*D*—H	H···*A*	*D*···*A*	*D*—H···*A*
C5—H5A···S1	0.93	2.70	3.1194 (16)	108
C15—H15A···N3^i^	0.93	2.48	3.343 (2)	155
C4—H4A···Cg1^ii^	0.93	2.62	3.5063	160

Symmetry codes: (i) *x*, *y*+1, *z*; (ii) −*x*+1, −*y*, −*z*+1.
